# Epithelial disruption: a new paradigm enabling human airway stem cell transplantation

**DOI:** 10.1186/s13287-018-0911-4

**Published:** 2018-06-13

**Authors:** Nigel Farrow, Patricia Cmielewski, Martin Donnelley, Nathan Rout-Pitt, Yuben Moodley, Ivan Bertoncello, David Parsons

**Affiliations:** 1 Robinson Research Institute, University of Adelaide, 55 King William Road, Adelaide, South Australia 5005 Australia; 2 Adelaide Medical School, University of Adelaide, Adelaide Health and Medical Sciences building, Corner of North Terrace and George Street, Adelaide, South Australia 5000 Australia; 3 Department of Respiratory and Sleep Medicine, Women’s and Children’s Hospital, 72 King William Road, North Adelaide, South Australia 5006 Australia; 4 Australian Respiratory Epithelium Consortium (AusRec), Perth, Western Australia 6105 Australia; 5 School of Medicine and Pharmacology, University of Western Australia, 35 Stirling Highway, Crawley, Perth, Western Australia 6009 Australia; 6 Lung Health Research Centre, Department of Pharmacology and Therapeutics, University of Melbourne, Level 8, Medical Building (No. 181) Map, Corner of Grattan Street and Royal Parade, Melbourne, Victoria 3010 Australia

**Keywords:** Stem cells, Airways, Transplantation, Gene therapy

## Abstract

**Background:**

Airway disease is a primary cause of morbidity and early mortality for patients with cystic fibrosis (CF). Cell transplantation therapy has proven successful for treating immune disorders and may have the potential to correct the airway disease phenotype associated with CF. Since in vivo cell delivery into unconditioned mouse airways leads to inefficient engraftment, we hypothesised that disrupting the epithelial cell layer using the agent polidocanol (PDOC) would facilitate effective transplantation of cultured stem cells in mouse nasal airways.

**Methods:**

In this study, 4 μL of 2% PDOC in phosphate-buffered saline was administered to the nasal airway of mice to disrupt the epithelium. At 2 or 24 h after PDOC treatment, two types of reporter gene-expressing cells were transplanted into the animals: *luciferase*-transduced human airway basal cells (hABC-Luc) or *luciferase*-transduced human amnion epithelial cells (hAEC-Luc). Bioluminescence imaging was used to assess the presence of transplanted *luciferase*-expressing cells over time. Data were evaluated by using two-way analysis of variance with Sidak’s multiple comparison.

**Results:**

Successful transplantation was observed when hABCs were delivered 2 h after PDOC but was absent when transplantation was performed 24 h after PDOC, suggesting that a greater competitive advantage for the donor cells is present at the earlier time point. The lack of transplantation of hAECs 24 h after PDOC supports the importance of choosing the correct timing and cell type to facilitate transplantation.

**Conclusions:**

These studies into factors that may enable successful airway transplantation of human stem cells showed that extended functioning cell presence is feasible and further supports the development of methods that alter normal epithelial layer integrity. With improvements in efficacy, manipulating the airway epithelium to make it permissive towards cell transplantation may provide another option for safe and effective correction of CF transmembrane conductance regulator function in CF airways.

## Background

Cystic fibrosis (CF) is an autosomal recessive genetic disorder that affects multiple organs, but the main cause of morbidity and early mortality is lung disease [1]. The cystic fibrosis transmembrane conductance regulator (CFTR) protein is a chloride channel on the surface of epithelial cells. Mutations in the *CFTR* gene result in the production of CFTR that does not function correctly or is not present at a sufficient quantity. The inability to transport chloride ions alters the osmotic balance in the airway lumen, and the airway surface liquid that lines the epithelial layer of the conducting airways is reduced [2]. The cilia that are a part of the epithelial architecture and are required to clear pathogens from the airway are immobilised in a milieu of highly viscous mucous [3]. This provides an airway environment amenable to colonisation by bacterial and viral pathogens. The lung disease that is associated with the CF airway phenotype is responsible for the roughly 40-year life expectancy of the patient with CF.

Correcting the root cause of the CFTR defect at the gene level has the potential of being an effective treatment for patients with all classes of CFTR mutation. One method by which CFTR function could be restored at a genetic level is via a cell therapy, in which CFTR-competent cells are transplanted into the airways. Here, we define transplantation as the act of delivery, lodgement and initial integration of regenerative donor cells into the airway epithelium, and engraftment is defined as the ability of those cells to subsequently proliferate and repopulate the airways with functionally differentiated progeny long-term. The archetypal example is bone marrow transplantation, in which both autologous and allogeneic haematopoietic stem cell transplantation have been successful in regenerating the immunohaematopoietic system of patients with life-threatening haematological and immunodeficiency disorders [4, 5]. Therapies using haematopoietic stem cell transplantation are now successfully performed in over 50,000 patients per year world-wide [6]. Lessons learnt in devising and refining immunohaematological cellular therapies point to the importance of factors that include the correct identification and selection of repopulating cells, the route of cell delivery, and the choice of pre-conditioning regimen [7]. With this background, a roadmap can be envisioned for the development of similar regenerative therapies able to correct intractable respiratory diseases such as CF.

In this study, we have assessed (1) the use of human airway basal cells (hABCs) and human amnion epithelial cells (hAECs) as potential donor cells, (2) the use of polidocanol (PDOC) as a model for an epithelial disrupting agent to condition the airway prior to donor cell transplantation and (3) the influence of the time interval between the pre-conditioning treatment and cell delivery on the ability to transplant donor cells.

## Methods

The first experiments were designed to assess the transducability of the two cell types (hABCs and hAECs) with a lentiviral (LV) vector containing the LacZ reporter gene. A separate group of cells were expanded, transduced with an LV vector containing the luciferase (Luc) transgene, and prepared for in vivo delivery. Transduced cells were delivered to normal mouse nasal airways after treatment with either a phosphate-buffered saline (PBS) sham control or PDOC by using two different intervals between epithelial disruption and cell delivery. Transplantation levels were assessed by bioluminescence imaging.

### Cell culture production of hABCs and hAECs

Human primary airway cells (human bronchial epithelial cells, or HBECs, cc-2540 s, Lonza, Mount Waverley, VIC, Australia) were seeded onto 25-cm^2^ collagen-coated flasks to isolate the hABC population. Cells were expanded by using Bronchial Epithelial Cell Growth Medium (BEGM, cc-3170, Lonza), and passaged twice. Samples were taken during passaging to confirm the basal cell identity by cytokeratin 5 (Krt5) staining as previously published [8].

hAECs were obtained from the Amnion Cell Biology Group, the Ritchie Centre, Hudson Institute of Medical Research, Monash University, Clayton, VIC, Australia. Cells were seeded onto 75-cm^2^ collagen-coated flasks, expanded and passaged once in Epi Growth Medium (215–500, Sigma-Aldrich, Sydney, NSW, Australia).

### Lentiviral vector production

VSV-G–pseudotyped HIV-1–based LV vectors expressing either nuclear-localised LacZ under transcriptional control of the MPSV promoter (LV-LacZ) or Luc with the ef1α promoter (LV-Luc) were produced in accordance with previously published methods [9]. The titre of the LacZ vector was 1.3 × 10^9^ Tu/mL for in vitro studies. The titre for the Luc vector used in cell transplantation studies was 6.8 × 10^8^ Tu/mL, as assayed by quantitative polymerase chain reaction [10].

### Assessment of in vitro LacZ transduction efficiency

In a previous short-term study, hABC cultures were transduced at multiplicities of infection (MOIs) of 0, 10 and 100, and the MOI of 10 produced close to 100% transduction [8]. To confirm that transgene expression was maintained in cells long-term, a subset of hABC-LacZ cultures (MOI 10) were grown for 4 weeks and processed for analysis of gene expression, and quantification was performed as previously described [8]. In the present study, the transduction efficiency of the same vector (LacZ) in hAECs at an MOI of 10 was assessed for comparison with transduction of hABCs. The hAEC cultures were seeded at a density of 2.5 × 10^5^ cells per well onto Nunc™ six-well plates coated with type 1 rat tail collagen. Two hours later, when 75% confluent and cells had adhered, they were treated with the LV vector, diluted with PBS as appropriate, at an MOI of 0 (PBS only) or 10. Cell cultures were incubated at 37 °C and 5% CO_2_ in a humidified chamber. One day after LV vector treatment, the media was aspirated, cells were washed with PBS at room temperature, fresh pre-warmed (37 °C) BEGM was added, and the cultures were returned to the incubator and maintained for 3 days. Quantification of LacZ gene expression was performed in accordance with previously published methods [8].

### Reporter gene transduction of hABCs and hAECs for in vivo delivery

hABCs and hAECs were grown on 75-cm^2^ collagen-coated flasks, expanded to 75% confluency, treated with the LV-Luc vector at the chosen MOI of 10, and incubated at 37 °C and 5% CO_2_ overnight in a humidified chamber. To confirm that cells expressed Luc prior to transplantation, a subset were examined by using bioluminescence imaging (IVIS Lumina XRMS, Xenogen Corporation, Alameda, CA, USA). Media was removed from the flasks, 2 mL of D-luciferin (30 mg/mL in PBS) was added, and imaging was performed. A circular region of interest (ROI) was used for quantification, and blank flasks containing no cells were imaged as controls.

On the following day, transduced cells (designated hABC-Luc and hAEC-Luc) were harvested by using HEPES-buffered saline, Trypsin/EDTA, and Trypsin neutralising solution (ReagentPack, cc-5034, Lonza) in accordance with the instructions of the manufacturer. Cells were re-suspended in PBS, cell counts were obtained, and 1.3 × 10^5^ cells per aliquot were held on ice for immediate delivery into mouse airways.

### Animal studies

All experiments were approved by the Women’s and Children’s Hospital and University of Adelaide animal ethics committees. Female C57BL/6 mice (6–8 weeks of age) were anaesthetised with an intraperitoneal (i.p.) injection containing a mixture of 10 μL/g body weight of medetomidine (Domitor, 0.1 mg/mL, Orion Corporation, Espoo, Finland) and ketamine (7.6 mg/mL, Parnell Laboratories, Alexandria, NSW, Australia). After each experimental procedure, anaesthesia was reversed with 1 μg/g body weight of atipamezole hydrochloride (Antisedan, Orion Corporation) also delivered as an i.p. injection, and mice were held in a humidified incubator until fully recovered. At termination of the animal studies, mice were humanely killed by 100% CO_2_ inhalation.

### Effect of PDOC treatment on nasal airway epithelium prior to cell transplantation

To assess the effect of PDOC on the epithelium at 2 and 24 h [11] post-delivery, the nasal airways of mice (*n* = 3) were exposed to 4 μL of 2% PDOC (3055–99-0, Sigma-Aldrich, St. Louis, MO, USA) in PBS. At either 2 or 24 h later, animals were humanely killed, relevant nasal tissue samples were collected and processed, and serial sections were examined histologically. Haematoxylin and eosin (H&E) staining was performed on 5-μm sections mounted on glass slides. Antibody staining for Krt5 was performed on adjacent 5-μm sections. After sodium citrate antigen retrieval, sections were stained with rabbit anti-Krt5 antibody (ab52635, Abcam, Melbourne, VIC, Australia; 1:350). The slides were placed in a humid incubation chamber and stored overnight at 4 °C. Goat anti-rabbit IgG H&L HRP (ab97051, Abcam; 1:500) was used as a secondary antibody for 1–2 h. An Abcam DAB Substrate Kit (ab64238) was applied for 10 min, and the colour development was stopped by rinsing tissue sections with PBS/0.05% Tween 20. Sections were counterstained with Meyer’s haematoxylin.

### In vivo cell transplantation

In the transplantation trials, the nasal airways were exposed to 4 μL of PBS or 2% PDOC in PBS delivered into the right nostril to disrupt the epithelium prior to cell delivery. At either 2 or 24 h after PDOC or PBS delivery, 3 × 10 μL aliquots of cells (hABC-Luc or hAEC-Luc) were delivered to the previously treated nasal passage at 10-min intervals between bolus aliquots.

### Bioluminescence imaging

One week after hABC-Luc or hAEC-Luc transplantation, mice were anaesthetised and Luc expression was assessed 10 min after a 50-μL intranasal bolus of D-luciferin (15 mg/mL in PBS). The nasal airways of treated mice were imaged by using the IVIS Lumina XRMS and auto-exposure with mice in a supine position. The resultant bioluminescent flux (photons/s) was measured after background image subtraction in a 1.5-cm^2^ square ROI in accordance with the instructions of the manufacturer (Igor Pro 4.09A Living Image Software, Xenogen Corporation). Mice were re-imaged at 3, 5 and 8 weeks after cell transplantation.

### Assessment of circulating luciferase antibodies

Blood was removed by submandibular puncture directly after each bioluminescence imaging event and via cardiac puncture after study termination. Blood was processed and circulating antibodies to the Luc protein were assessed as previously published [9].

### Detection of human cells in hABC-Luc– and hAEC-Luc–treated nasal airways

Mouse paraffin embedded nasal airway tissue sections were de-paraffinised and re-hydrated through sequential incubation twice in xylene for 3 min, twice in 100% ethanol for 2 min and 90% ethanol, 70% ethanol and milli-Q water for 2 min each. Antigen retrieval was carried out by placing sections in sodium citrate buffer (10 mM sodium citrate, 0.05% Tween-20, pH 6.0) at 90–100 °C for 20 min and allowed to cool to room temperature for 20 min. Sections were blocked with hydrogen peroxidase (ab64218, Abcam) for 10 min at room temperature, washed briefly twice in wash buffer (PBS/0.05% Tween-20) and blocked in blocking buffer (2% sheep serum, 0.1% bovine serum albumin, 0.1% gelatin, 0.1% Triton X-100 and 0.05% Tween-20) for 1 h at room temperature. Sections were washed twice in wash buffer and incubated with 1:1000 primary antibody to human mitochondria (Anti-Mitochondria, ab92824, Abcam) diluted in blocking buffer at room temperature for 1 h. Sections were washed with wash buffer and incubated with 1:400 secondary anti-body, Anti-Mouse IgG, Horseradish Peroxidase-Linked Species-Specific Whole Antibody (from sheep) (NA931, GE Life Sciences, ECL™, Parramatta, NSW, Australia) in blocking buffer for 1 h at room temperature. Sections were washed with wash buffer and secondary antibody was stained for 10 min with DAB substrate kit (ab64238, Abcam) (30 μL DAB Chromogen/1.5 mL DAB substrate) and washed with wash buffer. Sections were counterstained by rinsing in milli-Q water followed by Meyer’s haematoxylin for 90 s. Sections were washed in milli-Q water and sections were dipped into Scott’s tap water substitute for 5 s. Sections were de-hydrated by sequentially incubating the sections in milli-Q water, 70% ethanol, 90% ethanol, twice in 100% ethanol for 10 s each and xylene twice for 1 min, and a cover slip was added with mounting media. Slides were examined for the presence of human cells under 20× magnification.

### Statistics

Results are represented as a mean and standard error. Data were tested for normality assumptions and analysed by using GraphPad Prism 6 (GraphPad Software Inc., San Diego, CA, USA). Statistical significance was set at *P* = 0.05. Analysis of two treatment groups was performed by using a *t* test, and multiple treatment groups were analysed by two-way analysis of variance (ANOVA) with Sidak’s multiple comparison.

## Results

### Vector transduction of hABCs and hAECs

**Fig. 1:**
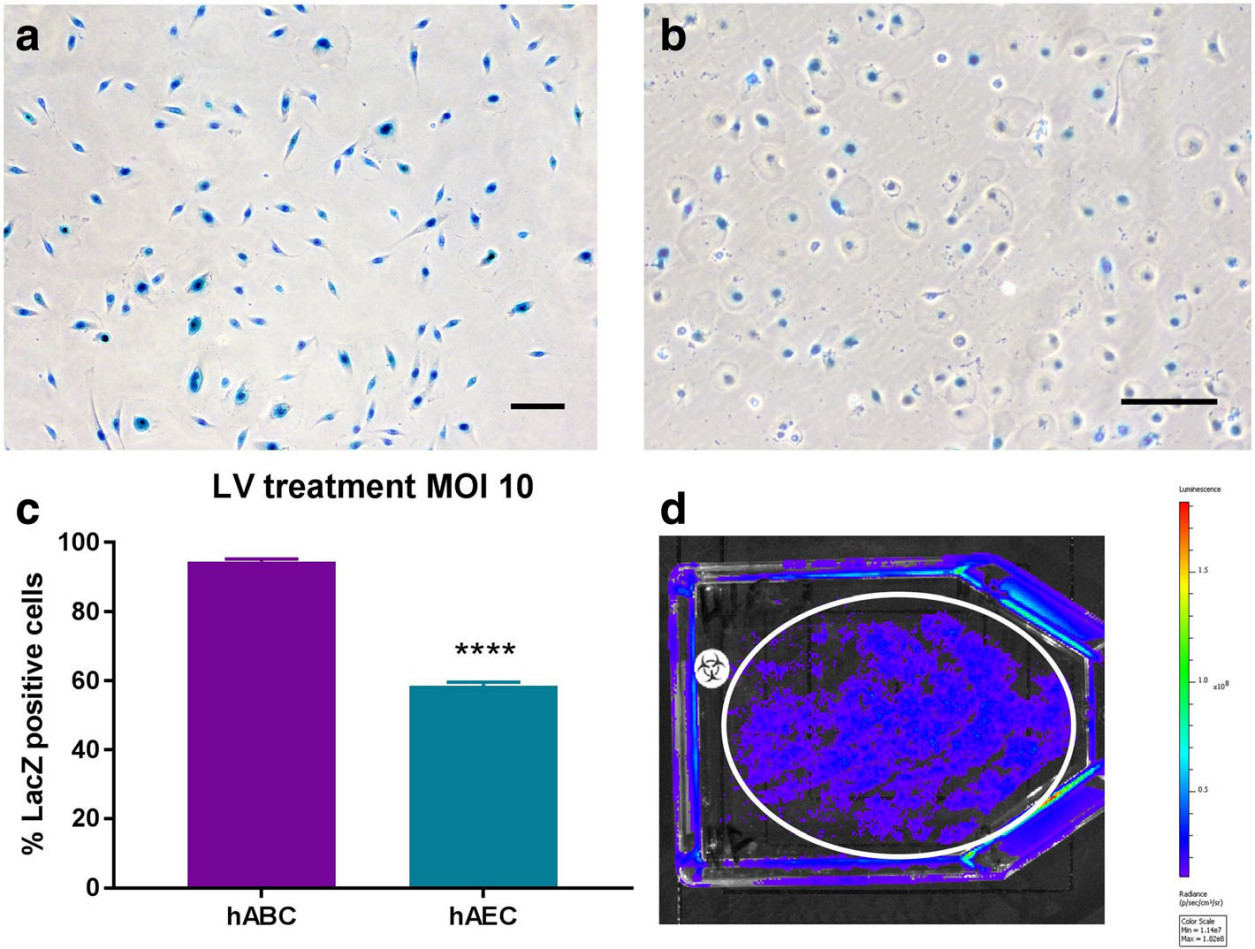
LV-LacZ reporter gene expression at an MOI of 10 in (**a**) hABCs and (**b**) hAECs. **c** In vitro LacZ transduction efficiency for hABCs compared with hAECs. ****P < 0.0001, *t* test, *n* = 3. **d** Luciferase reporter gene expression (circular region of interest) in hAEC cultures after LV-Luc vector treatment at an MOI of 10 shows that the cells were successfully transduced prior to use in the in vivo studies. Scale bars = 20 μm. *Abbreviations*: *hABC* human airway basal cell, *hAEC* human amnion epithelial cell, *LV-LacZ* HIV-1–based LV vectors expressing nuclear-localised LacZ under transcriptional control of the MPSV promoter, *MOI* multiplicity of infection

Prior to the reporter gene transduction procedures, hABC cultures were confirmed as 95% pure basal cells via Krt5 staining. hABCs and hAECs were expanded in culture and transduced with an LV vector carrying the LacZ or Luc reporter gene at an MOI of 10 (Fig. 1). hABC-LacZ were cultured for 4 weeks, and X-gal staining showed that the cells remained healthy and viable and that LacZ transgene expression was sustained for this period (Fig. 1a). To test the efficiency of the LV vector to transduce hAECs, cells were treated with MOIs of 0 (PBS control) and 10 (Fig. 1b). No LacZ expression was observed at an MOI of 0 (not shown). LacZ gene expression in the treated groups showed transduction efficiencies of 94.5% for hABCs compared with 58.5% for hAECs for an MOI of 10, *P* < 0.0001, *t* test, *n* = 3 (Fig. 1c).

A subset of the flasks were imaged prior to transplantation into the nasal airways of mice, confirming that hAEC-Luc cells expressed the Luc reporter gene (Fig. 1d). The resultant flux (9.03 × 10^9^ photons/s) observed in the T75 flask represented 50–70% transduction efficiency of the hAEC populations by circle ROI. No Luc was detected in the blank flask, confirming that the Luc-expressing cells were the source of the intense signal detected at the flask edges.

### PDOC treatment of nasal airway epithelium and initial cell transplantation

**Fig. 2:**
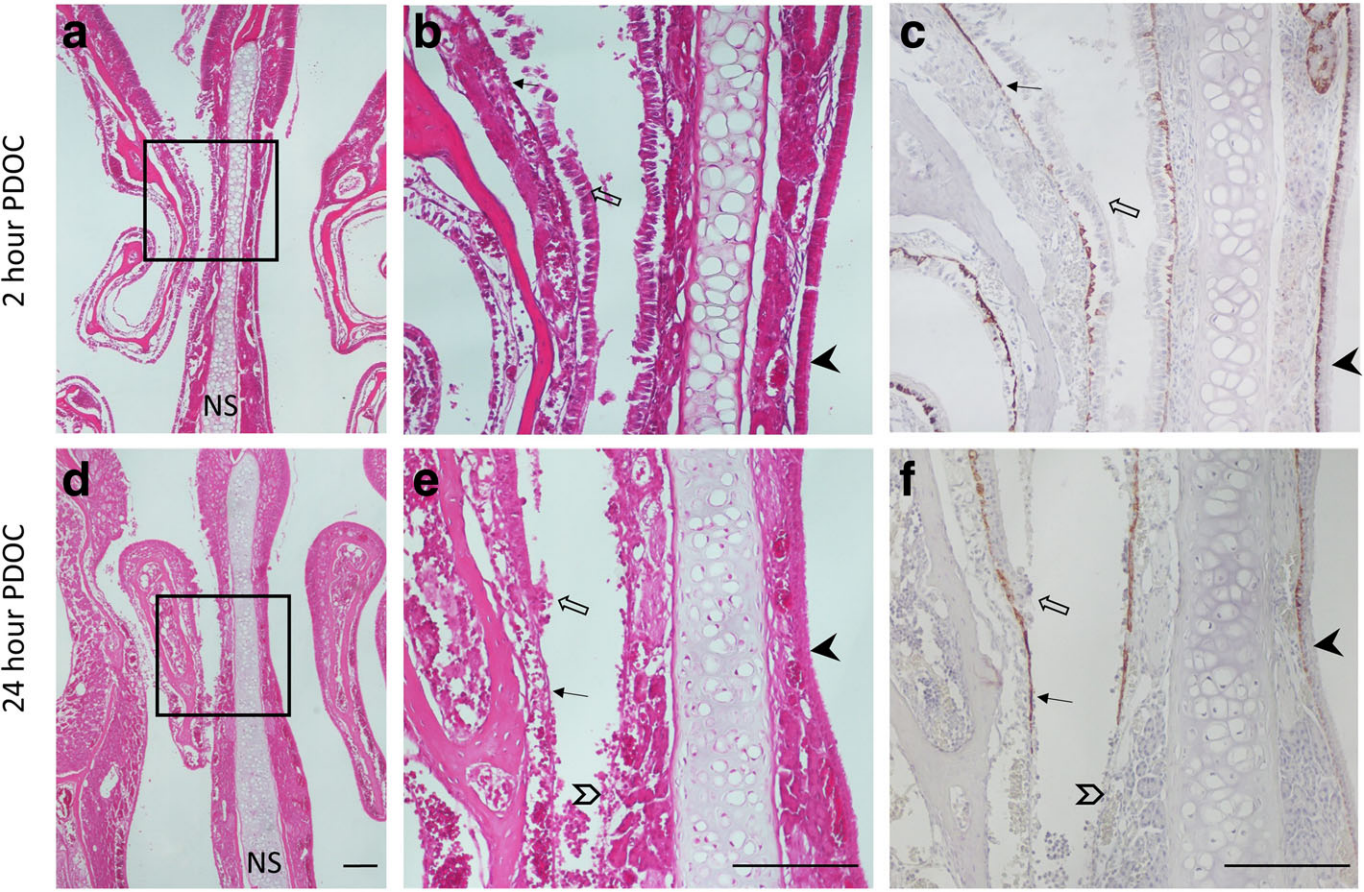
Histological images of the anterior mouse nose 2 or 24 h after PDOC treatment only; the septum (NS) separates the treated (*left*) and untreated control (*right*, *solid black arrowheads*) nasal airways. Regions of epithelial cell loss (*black arrows*), the presence of cell debris (*open arrows*), and a loss of basal cells and basal lamina integrity (*open arrowheads*) are apparent. Scale bars = 0.2 mm. Panels **b** and **c** are an enlargement of **a**; **e** and **f** are an enlargement of **d**. Panels **a**, **b**, **d**, and **e** are all H&E-stained; **c** and **f** are cytokeratin 5–stained serial sections from (**b**) and (**e**), respectively. *Abbreviations*: *H&E* haemotoxylin and eosin, *NS* not significant, *PDOC* polidocanol

Nasal airways treated with 4 μL of 2% PDOC showed that 2 h after treatment there were denuded portions of epithelium, disrupted areas of cells in the process of being removed, and considerable cell debris present (Fig. 2a, b). Twenty-four hours after treatment with 2% PDOC, large areas of epithelium were absent, leaving the basement membrane and basal cells intact (Fig. 2c, d). Small amounts of cellular debris were still present 24 h after PDOC treatment. Three unexpected deaths (of the 10 animals) occurred during hABC-Luc cell delivery conducted 2 h after PDOC. For this reason, the 2-h hAEC-Luc study was not initiated.

### Delivery of hABC-Luc to nasal airways 2 h after PDOC treatment

**Fig. 3:**
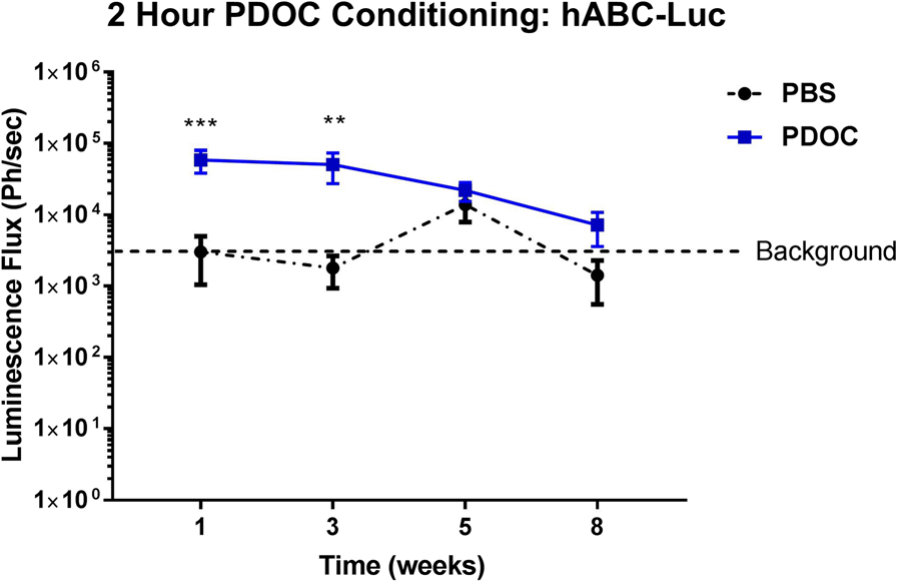
Luc expression was detected for at least 21 days in three out of seven mice that received hABC-Luc 2 h after the PDOC treatment (**P < 0.01, ***P < 0.001 two-way analysis of variance, Sidak’s multiple comparison, *n* = 7–10/group). *Abbreviations*: *hABC-Luc luciferase*-transduced human airway basal cells, *PBS* phosphate-buffered saline, *PDOC* polidocanol

**Fig. 4:**
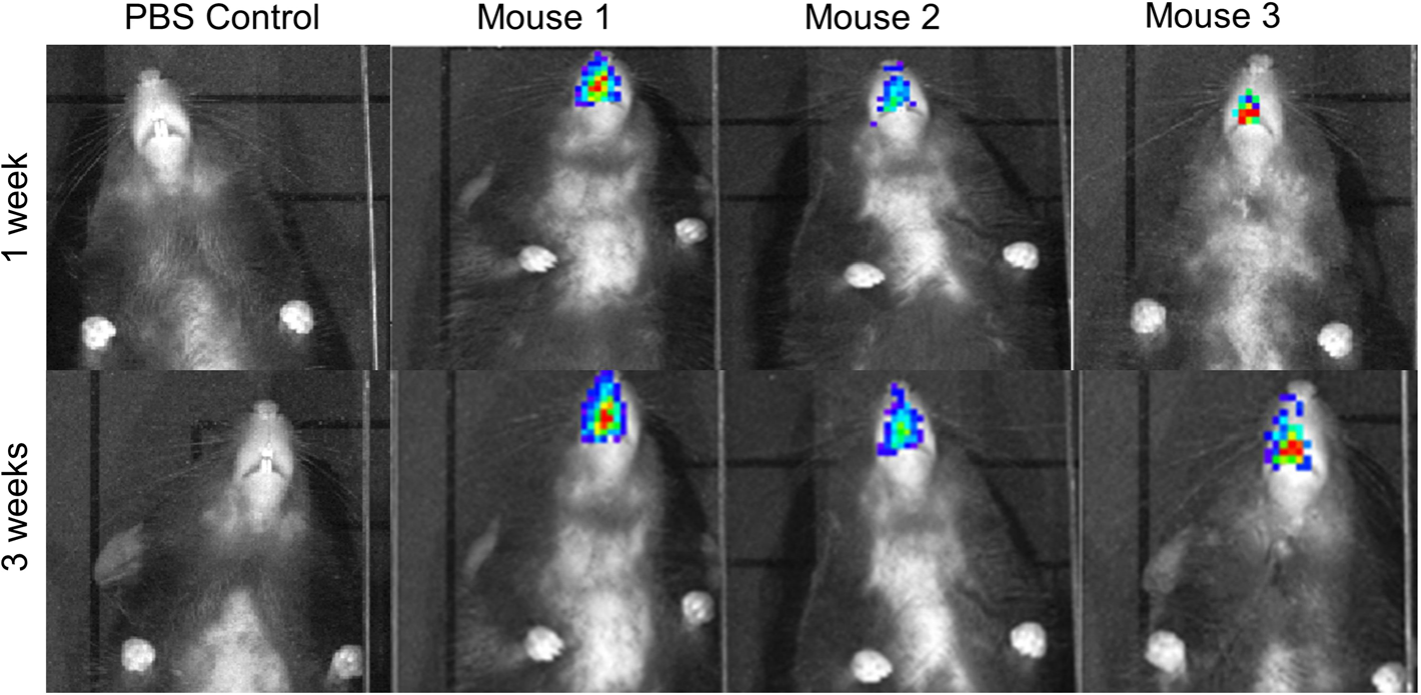
Bioluminescence in vivo imaging of hABC-Luc delivery revealed luciferase reporter gene expression in the nasal airways of three polidocanol (2 h conditioning)–treated mice and a PBS control mouse at 1 week and 3 weeks after delivery. *Abbreviations*: *hABC-Luc luciferase*-transduced human airway basal cells, *PBS* phosphate-buffered saline

Animals that received PBS pre-treatment showed no Luc gene expression at any time point after cell delivery (background ROI only, Fig. 3). In the group that received PDOC 2 h prior to cell delivery, three of the seven remaining animals showed Luc gene expression at the 1- and 3-week time points (Figs. 3 and 4) that was significantly increased compared with PBS-conditioned mice (*P* < 0.001 at 1 week and *P* < 0.01 at 3 weeks, two-way ANOVA, Sidak’s multiple comparison, *n* = 7–10/group), and expression declined at the 5-week time point to reach baseline levels (background ROI) by the final (8-week) time point.

### Delivery of HABC-Luc or hAEC-Luc to nasal airways 24 h after PDOC delivery

**Fig. 5:**
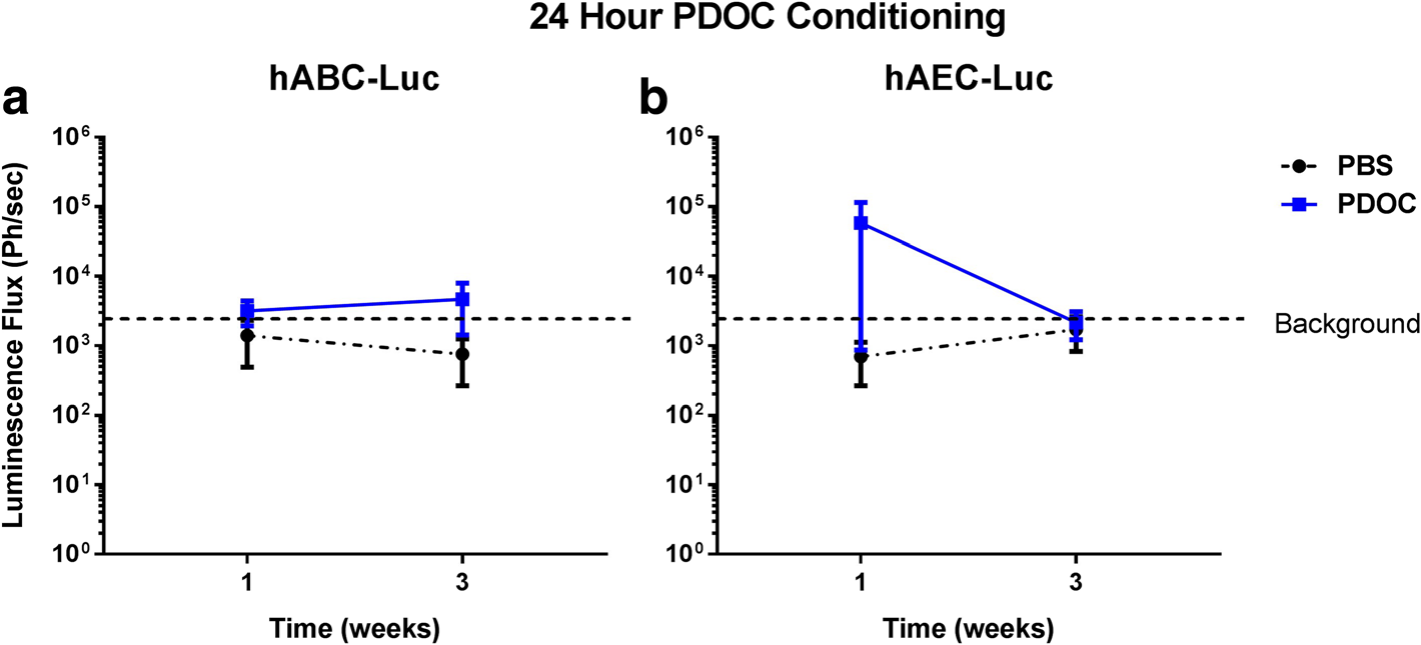
There was no significant difference in Luc reporter gene expression in the nasal airways at either 1 or 3 weeks after (**a**) hABC-Luc or (**b**) hAEC-Luc delivery 24 h after PDOC disruption, compared with PBS (not significant, two-way analysis of variance, Sidak’s multiple comparison, *n* = 5/group). *Abbreviations*: *hABC-Luc luciferase*-transduced human airway basal cells, *hAEC-Luc luciferase*-transduced human amnion epithelial cells, *PBS* phosphate-buffered saline, *PDOC* polidocanol, *Ph/sec* photons per second

Bioluminescence imaging showed that no gene expression was present in animals at either 1 week or 3 weeks after delivery of hABC-Luc 24 h after PDOC or PBS conditioning (Fig. 5a). One animal that received hAEC-Luc cells showed Luc expression 1 week after treatment, but no animals displayed Luc gene expression at 3 weeks (Fig. 5b), so the study was terminated at this point.

### Assessment of circulating antibodies to luciferase

Antibodies to Luc in blood serum samples taken prior to cell transplantation and at each bioluminescence imaging time point showed an absence of circulating antibodies above background levels (data not shown).

### Immunohistochemistry to assess the presence of transplanted cells

At the conclusion of the studies (3- and 8-week time points), when Luc expression was below the limit of detection, the epithelium appeared normal and comparable to a regrown epithelium seen after PDOC use in other studies [8, 11]. No human mitochondria were detected in the re-grown mouse nasal airway tissue, indicating that no hABC-Luc or hAEC-Luc cells were present, regardless of conditioning treatment and timing (data not shown).

## Discussion

A cell therapy to transplant a CFTR-competent cell population into the airways of patients with CF has the potential to correct the disease by restoring epithelial CFTR function and returning the airway environment to normal.

The premise of this mode of CF genetic therapy is that it would be able to prevent the onset of lung disease in young patients or halt disease progression in older patients with existing disease. Delivering a population of cells with stem or progenitor qualities would allow those cells to differentiate into the full range of cell types of the airway epithelium. This approach potentially has the advantage of providing an ongoing source of CFTR-expressing surface cells via the cell turnover process that is naturally present to deal with the replacement of ageing or damaged epithelial cells.

This study provides proof-of-principle that transplantation of healthy cells in the airways is feasible with the potential to alleviate CF-related airway disease and other airway diseases such as primary ciliary dyskinesia. We found that, when the airway epithelium was denuded by PDOC treatment, hABCs delivered into the denuded airways in mice were successfully transplanted and remained viable for 3 weeks after transplantation.

The quality and longevity of transplantation we observed were consistent with early results from experimental haematologic disease models, which revealed that the level and longevity of transplantation were inversely correlated with the proportion of host stem cells remaining after myeloablation. This means that the competitive advantage of the host cell is reduced by the specific cytotoxic regimen chosen to ablate endogenous stem cells and provides the transplanted donor cells with a competitive advantage [12].

Obtaining high levels of cell transplantation into the airway epithelium is clearly a challenge [13], and here we have demonstrated that donor cells fail to transplant in the absence of epithelial disruption prior to cell delivery. A recent study also showed that engraftment of transplanted human epithelial progenitors in a mouse model is dependent on epithelial ablation [14]. In that study, bleomycin was used to selectively target alveolar epithelial cell lineages in the distal lung; it also reported on preliminary translational studies for bronchiectasis, said to show clinical benefit in two patients. However, the mechanism for benefit in those studies is unclear.

PDOC treatment denudes the nasal or tracheal epithelium but spares basal cells [11], the cell group recognised as the stem cells of the upper airway [15]. Airway basal cells function to maintain epithelial integrity and homeostasis, through cell differentiation to repair the natural turnover of airway cells as well as epithelial injury. The difference in transplantation success from hABC delivery at 2 h after PDOC, compared with an absence of transplantation at 24 h, suggests that a greater competitive advantage for the donor cells is present at the earlier time point. The lack of efficacy to transplant hAECs at 24 h after PDOC supports the significance of correct timing to facilitate transplantation.

Denuding portions of airway with PDOC activates the resident basal cells to repair the epithelium [8], and exogenous donor cells given at 2 h after PDOC treatment will be in immediate competition with host cells for available transplantation sites [16] as well as in competition with host cells undertaking differentiation and repair of the damaged epithelium [16]. When transplanted, the donor cells introduced early into the damaged environment will likely be exposed to the same cues as the endogenous basal cells. However, if introduced later (e.g., as here, at 24 h), the donor cells may miss the cues to become involved in the repair process and so be unable to successfully transplant or engraft as they are outcompeted by the endogenous hABCs. Therefore, this study confirms that the quality of transplantation is dependent on both the ablative modality and the timing of donor cell delivery.

The assessment of circulating luciferase antibodies in blood serum taken prior to cell transplantation and when bioluminescent imaging was performed showed that no active immune response was detected. This finding indicates that the eventual loss of luciferase expression at 3 weeks was unlikely to be due to an immune response elicited by the presence of the foreign luciferase protein. It is possible that a recipient immune response to the hABCs themselves and not the luciferase transgene caused the disappearance of the Luc-transduced hABCs. For example, early allogeneic haematopoietic cell transplantation studies proposed recipient immune response(s) as a potential mechanism for graft failure in humans and that this might be overcome by more intensified conditioning, increased cell dose, or more effective immunosuppression [17]. More recently, mouse studies of naphthalene pre-conditioning of the airway epithelium prior to human cell transplantation also concluded that immunosuppression protocols might improve transplantation in allogeneic recipients [18].

A weakness of the study is that it was designed to assess the time course/longevity of potential transplantation and so animals could not be used to examine the transplantation process histologically. Hence, at study termination, when luciferase expression could no longer be detected, histological assessment could not demonstrate the presence of transplanted cells presence and differentiation. Although the deaths of three mice in the study is a concern, the positive Luc imaging results clearly validate the potential for the method, and future studies are warranted to assess whether lower doses of PDOC or the timing of cell delivery can overcome this limitation.

## Conclusions

In summary, this study shows that human airway basal stem cells can be transplanted into live (mouse) airway if the epithelium is first denuded or damaged. Our studies also provide an in vivo model suited to seeking the optimal methods to target cellular therapies for the treatment of CF lung disease and to identify the most appropriate regenerative cells suited to that purpose.

## Abbreviations

ANOVA: Analysis of variance
BEGM: Bronchial Epithelial Cell Growth Medium
CF: Cystic fibrosis
CFTR: Cystic fibrosis transmembrane conductance regulator
hABC: Human airway basal cell
hABC-Luc: *Luciferase*-transduced human airway basal cells
hAEC: Human amnion epithelial cell
hAEC-Luc: *Luciferase*-transduced human amnion epithelial cells
i.p.: Intraperitoneal
Krt5: Cytokeratin 5
LV: Lentiviral
LV-LacZ: HIV-1–based LV vectors expressing nuclear-localised LacZ under transcriptional control of the MPSV promoter
LV-Luc: HIV-1–based LV vectors expressing nuclear-localised Luc under transcriptional control of the ef1α promoter
MOI: Multiplicity of infection
PBS: Phosphate-buffered saline
PDOC: Polidocanol
ROI: Region of interest
